# Dose‐Escalation Regimens for Incretin Mimetics in Type 2 Diabetes Are Associated With Tolerance for Nausea and Vomiting

**DOI:** 10.1111/dom.70613

**Published:** 2026-02-27

**Authors:** Michael A. Nauck, Viktoria Punov, Yu Mi Kang, Soo Lim

**Affiliations:** ^1^ Diabetes, Endocrinology, Metabolism Section Medical Department I Josef‐Hospital, Ruhr University Bochum Bochum Germany; ^2^ Institute for Clinical Chemistry and Laboratory Medicine, University Medicine Greifswald Greifswald Germany; ^3^ Division of Endocrinology, Diabetes and Hypertension and TIMI Study Group, Brigham and Women's Hospital, Harvard Medical School Boston Massachusetts USA; ^4^ Department of Internal Medicine Seoul National University College of Medicine, Seoul National University Bundang Hospital Seongnam South Korea

**Keywords:** GLP‐1 analogue, pharmaco‐epidemiology, type 2 diabetes, weight control

## Abstract

**Aims/Hypothesis:**

Initial dose‐escalation has been shown to mitigate gastrointestinal adverse events in people treated with incretin‐based medications. We aimed to analyse dose‐response relationships for the proportion of study participants reporting nausea and vomiting among those exposed to incretin mimetics approved for Type 2 diabetes (GLP‐1 receptor agonists and the dual GIP/GLP‐1 receptor agonist tirzepatide) in Phase 1 (no or short dose escalation) and Phase 3 (with dose escalation) trials.

**Methods:**

Non‐linear regression analysis (curve fitting) was used to estimate the dose that would elicit nausea or vomiting in 50% of exposed subjects (ED_50_). We used the ratio of ED_50_ies determined for Phase 1 and Phase 3 trials as an indicator of developing tolerance.

**Results:**

The development of tolerance for nausea and vomiting (indicated by a ratio of ED_50_ies Phase 3/Phase 1) for semaglutide (both s.c. and oral) and for tirzepatide was significantly higher than 1. Comparing all approved incretin‐based medications, a higher ratio, indicating the development of more tolerance, was associated with (a) a longer drug escalation period and (b) a greater number of dose‐escalation steps. This ED_50_ ratio (Phase 3/Phase 1) was also significantly associated with effect sizes for intended therapeutic actions of incretin mimetics (reductions in HbA_1c_ and body weight).

**Conclusions/Interpretation:**

Taken together, optimised dose‐escalation regimens may lead to greater tolerance for nausea and vomiting, which allows to use of higher doses, which are associated with greater therapeutic effectiveness.

## 1 Introduction

Incretin mimetics are glucose‐lowering medications for the treatment of Type 2 diabetes, originally developed based on the glucose‐ and body‐weight‐lowering effects of glucagon‐like peptide‐1 (GLP‐1). In this class, there are the short‐acting compounds exenatide b.i.d. and lixisenatide, the long‐acting compounds liraglutide (once daily injection), exenatide once weekly, dulaglutide, albiglutide and semaglutide (once weekly injection) and oral semaglutide [1, 2, 3, 4], and the dual glucose‐dependent insulinotropic polypeptide (GIP)/GLP‐1 receptor agonist tirzepatide [4, 5, 6]. Tirzepatide treatment induces larger reductions in HbA_1c_ or body weight than does semaglutide, a potent GLP‐1 receptor mono‐agonist [7].

A series of head‐to‐head comparisons [7, 8, 9, 10, 11, 12, 13, 14, 15, 16, 17] and network meta‐analyses [18, 19] have consistently suggested that the effect sizes elicited for HbA_1c_, fasting plasma glucose and body weight reductions vary considerably across the currently approved incretin mimetics among people with Type 2 diabetes. This has largely been attributed to differences in molecular structure and pharmacokinetic behaviour, characteristics of their interaction with GLP‐1 (and GIP) receptors [2, 3], and the selection of doses for Phase 3 trials and approval that provide an appropriate balance of efficacy and risk for adverse events.

Incretin mimetics share a common adverse event profile: This includes nausea, vomiting, diarrhoea and other abdominal symptoms, often referred to as gastrointestinal side effects. These symptoms mainly occur immediately after initiating treatment or after increasing the dosage as part of a recommended dose‐escalation regimen [20]. As such, recommendations suggest to start with low doses, and then to slowly increase the exposure, usually in multiple steps, allowing time to develop some tolerance [21].

We have recently reported a systematic analysis of Phase 3/4 clinical trials from our group that has compared pooled (placebo‐subtracted) effect sizes for intended therapeutic actions (reductions in HbA_1c_ and body weight) for all approved GLP‐1 receptor agonists and the GIP/GLP‐1 dual receptor agonist tirzepatide (Table S1), and has also captured the proportions of patients reporting nausea, vomiting and other gastrointestinal (GI) adverse events for each compound/preparation and dose [22]. This study documented substantial differences in efficacy between available incretin mimetics, while, at the highest approved doses, the risk for adverse events like nausea and vomiting was generally similar (when expressed as the odds ratio for the proportion of study participants reporting nausea or vomiting with active treatment at the highest approved dose vs. placebo treatment) [22].

The process of developing gastrointestinal tolerance, when initiating treatment with GLP‐1 receptor agonists or tirzepatide, has not been well described or even systematically analysed. Empirically, over the time from introducing the first incretin mimetic drug, exenatide b.i.d. (2006) until the recent approval of tirzepatide (2022), the dose escalation strategies in clinical trials have changed considerably with respect to their duration (longer), the number of dose steps (more), and comparing the initial dose with the intended highest maintenance dose (lower) (Table 1, Figure S1). Therefore, this formed our central hypothesis that differences in effect sizes for intended therapeutic actions between incretin mimetics may reflect differences in the efficacy of various dose‐escalation regimens to elicit tolerance for nausea and vomiting. If so, a greater degree of tolerance achieved (responsible for less side effects during the maintenance period of treatment) would the exposure to higher doses, which in turn can explain greater effectiveness. Therefore, this study was designed to compare dose–response relationships for eliciting nausea and vomiting in Phase 1 clinical trials (usually without enough time or other measures to induce tolerance) and Phase 3/4 (allowing the development of tolerance to the degree achieved with each specific dose‐escalation regimen).

**TABLE 1:** Characteristics of the recommended dose‐escalation regimens for GLP‐1 receptor agonists and the GIP/GLP‐1 dual receptor agonist tirzepatide.

Compound/preparation	Initial dose/frequency of administration	Next and following dose steps	Time before increasing the dose	Highest maintenance dose	Duration of up‐titration (weeks)	Number of dose steps	Initial dose (proportion of maintenance dose) (%)
Exenatide b.i.d.	5 μg twice daily	10 μg twice daily	4 weeks	10 μg twice daily	4	2	50
Lixisenatide	10 μg once daily	20 μg once daily	2 weeks	20 μg twice daily	2	2	50
Liraglutide	0.6 mg once daily	1.2, then 1.8 mg per day	1 week	1.8 mg daily	2	3	33.3
Exenatide q.w.	2 mg per week	No dose escalation	n.a.	2 mg per week	n.a.	1	100
Dulaglutide	1.5 mg per week	No dose escalation	n.a.	1.5 mg per week	n.a.	1	100
Albiglutide	30 mg per week	50 mg per week (if needed)	Variable	30 or 50 mg per week	Variable	1–2	60 or 100
Semaglutide s.c.	0.25 mg per week	0.5, then 1.0 mg per week	4 weeks	1.0 mg per week	8	3	25
Semaglutide oral	3 mg per day	7 mg per day, then 14 mg per day	4 weeks	14 mg per day	8	3	21.4
Tirzepatide	2.5 mg per week	5 mg, then 7.5 mg, then 10 mg, then 12.5 mg, finally 15 mg per week	4 weeks	15 mg per week	20	6	16.7

*Note*: Data are from the pivotal clinical trial programs and current recommendations as per medication package inserts.

## 2 Methods

### 2.1 Design of the Analysis

Based on results from a recent meta‐analysis comparing (placebo‐subtracted) efficacy and GI tolerability of approved incretin‐based medications in T2D [22], dose–response relationships for the proportion of study participants reporting nausea or vomiting in Phase 3/4 clinical trials were captured. The proportion of study participants reporting nausea and vomiting in Phases 1 (studied in healthy people or in populations with Type 2 diabetes) and 2 (all in people with Type 2 diabetes) trials with any studied dose of the same incretin mimetics was extracted from all available studies (Tables S2 and S3). Dose–response relationships were analysed by curve‐fitting, with the aim to estimate, for each compound/preparation separately, the dose that would elicit nausea or vomiting in 50% of the population (ED_50_). A higher ED_50_ in Phase 3 than in Phases 1 or 2 would indicate the development of tolerance.

### 2.2 Analyses of Dose–Response Relationships for Nausea and Vomiting

**FIGURE 1:**
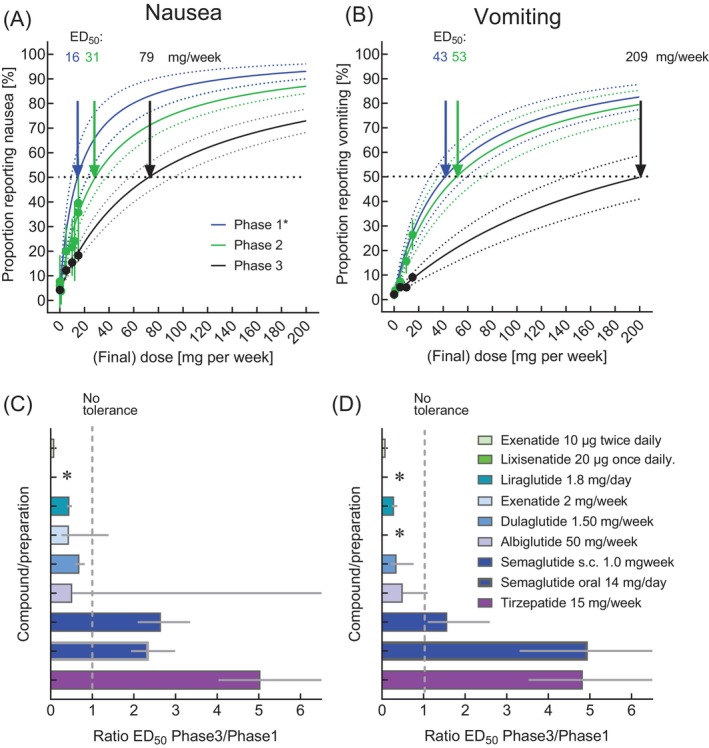
Evidence of the development of tolerance for nausea/vomiting by analysing dose–response relationships and estimating (by curve fitting) the dose of tirzepatide, which would elicit nausea or vomiting in 50% of the populations studied (ED_50_), and results of such analyses for all GLP‐1 receptor agonists and the dual GIP/GLP‐1 receptor agonist tirzepatide from clinical trials ranging from Phase 1 to Phase 3. Dose–response relationship for tirzepatide and nausea (A) and vomiting (B); data points are from Phases 1, 2 and 3 are shown with blue, green and black symbols, respectively. The dose–response relationships (same colours) derived by curve fitting are shown with 95% confidence bands (dotted lines). Development of tolerance as indicated by the ratio of estimated ED_50_ies for Phase 3 divided by the ED_50_ies for Phase 1 regarding nausea (C) and vomiting (D) for exenatide b.i.d., lixisenatide, liraglutide, exenatide once weekly, dulaglutide, albiglutide, semaglutide (for subcutaneous and oral administration) and tirzepatide. Data from Phase 3 trials have been taken from a recent systematic analysis and show pooled data from for each approved dose from placebo‐controlled Phase 3/4 studies [22]. Data regarding Phases 1 and 2 studies are from publications summarised in Tables S2 and S3. Table S4 summarises the estimated ED_50_ies for nausea and vomiting, from which the ratios (Phase 3 over Phase 1) displayed in panels (C) and (D) have been calculated, which serve as a measure of tolerance developing with dose‐escalation regimes.

For each compound/preparation (Table S1) and study phase (1, 2, 3/4), dose–response relationships for the proportion of study participants reporting nausea or vomiting were defined by non‐linear regression analysis (curve fitting) for any dose that had been tested. The equation used was ‘[agonist] vs. response (three parameters)’:

$$Y=\text{bottom}+\frac{\left(\mathrm{top}‐\text{bottom}\right)\cdot x}{{\mathrm{ED}}_{50}+x}$$

with the following definitions: The ‘bottom’ value was defined as the pooled results with placebo; the ‘top’ (maximum possible) value was chosen as 100%, since in Phases 1 and 2, some individual values were up to 79% [23, 24, 25], indicating that at a high‐enough dose, nausea could potentially be achieved in 100% of a given population. Input variables were the proportion (%) of *N* subjects reporting nausea or vomiting, the total number of subjects studied at each dose (*n*), and the standard error of the mean (SEM) calculated by standard equations from *N* (the number of subjects reporting nausea or vomiting) and *n* (the total number of subjects exposed to this dose of the compound). Curve‐fitting was performed under the following conditions: *outliers*: no special handling of outliers; *curve‐fitting method*: Least‐squares regression; *weighting*: no weighting; the sum‐of‐squares of the distances of the data points from the curve was minimised; convergence criteria: medium (automatically switching to strict convergence when needed); *replicates*: account for the *N* and scatter among replicates (from the respective standard error of the mean). Apart from defining the full dose–response relationship across all doses tested, one additional output was the estimated ED_50_ and its 95% confidence interval. Examples of such analyses are shown in Figure 1A,B. It should be noted that data points representing results from Phases 2 and 3 fall on the regression curve, but that the estimated ED_50_ is derived from a part of the regression curve far right from the area, where data points are located.

### 2.3 The Ratio of Estimated Doses Associated With Nausea or Vomiting in 50% of Patients (ED_50_ ) in Phase 1 and Phase 3/4 Clinical Studies as an Indicator of Tolerance

Estimated ED_50_ies for each compound/preparation for clinical trials from Phases 1, 2 and 3/4 and their 95% confidence intervals were compiled (Table S4) and compared. The ratio of ED_50_ies determined from Phases 3/4 and 1 and its 95% confidence interval was calculated. A higher ratio indicates that higher doses are necessary to elicit nausea or vomiting in 50% of the study populations and serves as a measure of developing tolerance after the dose‐escalation regimen typically recommended (and tested in the Phase 3 study programme) for each individual incretin mimetic.

### 2.4 Statistical Analyses

Significant differences between estimated ED_50_ were compared between Phase 1 and Phase 3/4 trials were defined as having non‐overlapping 95% confidence intervals. The significance of the development of tolerance for nausea or vomiting was determined by whether the lower bound of the 95% confidence interval of the ratio of ED_50_ies (Phase 3/4 over Phase 1) was higher than 1. These criteria indicated *p* values < 0.05. Curve‐fitting (non‐linear and linear regression analyses) were performed with GraphPadPrism 10 for Windows 64‐bit, version 10.6.1 (892) (Boston, MA, USA; www.graphpad.com). Results of linear regression analysis were expressed as the regression equation, the *r* value squared, the associated *p* value. *p* values < 0.05 were considered statistically significant.

## 3 Results

### 3.1 Dose‐Escalation Regimens for Incretin Mimetics in Type 2 Diabetes

Table 1 and Figure S1 summarise the characteristics of dose‐escalation regimens for individual incretin mimetics and their evolution over time. More recently developed incretin mimetics are initiated at lower initial doses relative to their intended maintenance doses. In addition, a clear trend is observed toward dose escalation occurring over a greater number of steps (overall range: 1–6), distributed across a substantially longer period of time (overall range: 0–20 weeks).

### 3.2 Multi‐Step Dose‐Escalation Regimens and Tolerance for Gastrointestinal Adverse Events

Because clinical trials conducted in drug development Phases 1, 2 and 3/4 are characterised by progressively longer durations, they allow little to no dose‐escalation in Phase 1, intermediate dose‐escalation in Phase 2, and more extensive dose‐escalation in Phase 3. Accordingly, we compared dose–response relationships for the proportion of exposed participants reporting nausea or vomiting across these phases. The incretin mimetics were tentatively be divided into three groups: (a) agents without or with minor dose‐escalation (i.e., exenatide once weekly, dulaglutide and albiglutide); (b) earlier‐approved compounds/preparations with short dose‐escalations periods lasting 2–4 weeks (i.e., short‐acting exenatide b.i.d. and lixisenatide, and long‐acting liraglutide) and (c) more recently developed incretin mimetics (semaglutide for s.c. and oral use, and tirzepatide), for which more elaborate dose‐escalation regimens have been developed (duration 8–20 weeks, 3–6 dose steps; Table 1).

### 3.3 Higher ED_50_ies After Elaborate Dose Escalation (Phase 3) as Compared to Phase 1

**FIGURE 2:**
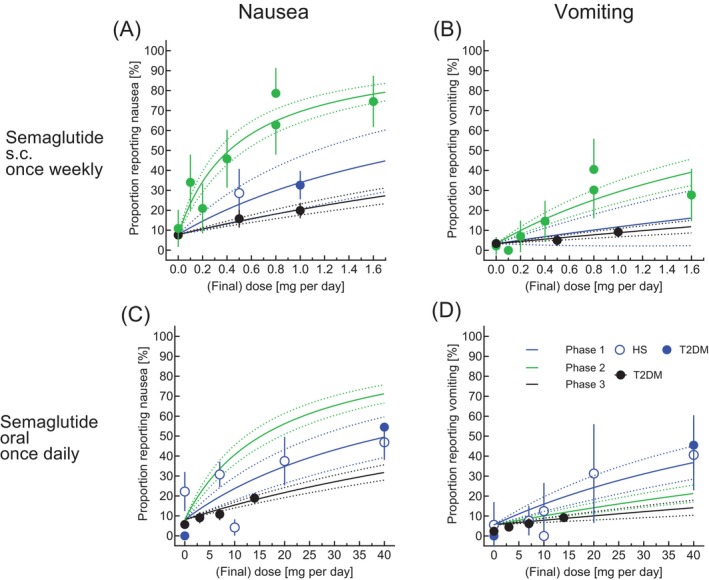
Evidence for the development of tolerance against nausea and vomiting with the GLP‐1 receptor agonists semaglutide (for subcutaneous injection and for oral use) based on a comparison of dose–response relationships reported from Phases 1, 2 and 3 clinical trials with different duration and dose‐escalation periods. Final doses (attained after dose‐escalation) of incretin mimetics (*x*‐axis) are plotted against the reported proportions of subjects reporting nausea (left hand panels, A, C) and vomiting (right hand panels, B, D) in Phase 1 studies (blue; healthy subjects: open symbols; Type 2‐diabetic subjects; filled symbols), Phase 2 (green) or Phase 3 (green). Dose response‐relationships and their 95% confidence bands derived from curve‐fitting are shown as dotted lines in the same colours. Data regarding Phases 1 and 2 studies are from publications summarised in Tables S2 and S3. Table S4 summarises the estimated ED_50_ies for nausea and vomiting from Phases 1, 2 and 3/4.

Our analysis is illustrated in Figure 1: Dose–response relationships for nausea (Figure 1A) and vomiting (Figure 1B) in Phases 1, 2 and 3 clinical trials indicate the highest burden of adverse events in Phase 1 studies, with the dose–response curves progressively shifting downward and to the right in Phase 2 and, more prominently, in Phase 3 in trials of tirzepatide. This pattern was also observed in Figure 2, which is restricted to the dose ranges evaluated in clinical trials (whereas Figure 1 also displays results of non‐linear regression analysis extrapolated into the higher dose range). Taken together, these findings indicate a significantly higher ED_50_ for both nausea and vomiting in Phase 3 compared with Phase 1 for tirzepatide, as well as for subcutaneous and oral semaglutide (Figure 2).

### 3.4 Increased ED_50_ Ratios for Nausea and Vomiting in Phase 3 Versus Phase 1 Studies of Tirzepatide and Subcutaneous or Oral Semaglutide

Estimated ED_50_ for reporting nausea and vomiting across Phases 1, 2 and 3/4 and all approved compounds/preparations within the incretin mimetic class are reported in Table S4. Compared to the agents that were developed earlier, the ED_50_ ratios were higher for more recently developed compounds/preparations (i.e., subcutaneous and oral semaglutide and tirzepatide). The ED50 ratios consistently exceeding 1 from Phase 3 and Phase 1 indicates a significantly higher tolerance associated with more elaborate dose‐escalation regimens (Figure 1C,D; Figure 2). On the contrary, this was not the case for exenatide b.i.d., lixisenatide, liraglutide, exenatide once weekly, dulaglutide and albiglutide (Table S4, Figures S2 and S3) reflecting less development of tolerance for nausea or vomiting (Figure 1C,D).

### 3.5 ED_50_ Ratios Are Associated With Characteristics of Dose‐Escalation Regimens

**FIGURE 3:**
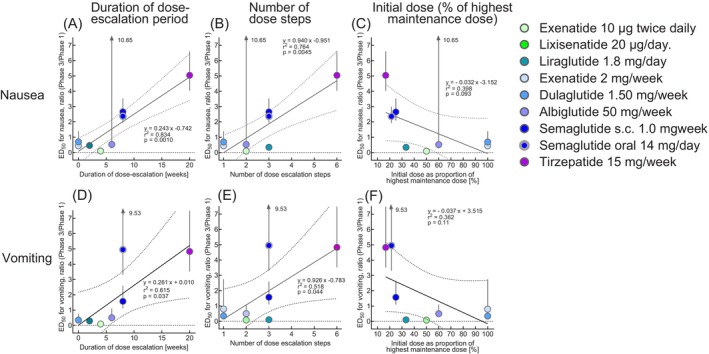
Relationship of characteristics of approved dose‐escalation regimens (duration of dose‐escalation, number of dose escalation steps and the initial dose as a proportion of the highest maintenance doses) for GLP‐1 receptor agonists and the dual GIP/GLP‐1 receptor agonist tirzepatide to the degree of tolerance against developing nausea and vomiting developed as the result of dose‐escalation with the regimens typical for exenatide b.i.d., lixisenatide, liraglutide, exenatide q.w., dulaglutide, albiglutide, semaglutide s.c., semaglutide p.o. and tirzepatide. Tolerance is quantified by comparing dose–response relationships for the development of nausea and vomiting between Phase 1 and Phase 3 based on the ratio of estimated effective doses_50%_ (ED_50_) from Phase 1 and Phase 3 studies (and their 95% confidence intervals) as determined by curve‐fitting (see Table S4). Statistical analysis: Linear regression analysis, reporting the regression equation, *r* ^2^ and the related *p* value. For details of characteristics (number of dose escalation steps, duration of dose escalation period and the initial dose as a proportion of the highest maintenance doses) of approved dose‐escalation regimens, see Table 1.

As shown in Figure 3, a linear regression analysis indicated a significant association between the duration of dose escalation periods and the number of dose escalation steps with the development of tolerance for both nausea and vomiting, as indicated by the respective ED_50_ ratios derived from dose–response relationships in Phase 3 versus Phase 1 studies (Figure 3A,B,D,E). A similar trend (not significant) was observed for the initial doses expressed as a proportion (percentage) of the intended highest maintenance dose (after dose escalation; Figure 3C,E), as well as for the duration of dose escalation periods (estimated from published population pharmacokinetics for each compound/preparation; Table S5). Time to reach steady‐state drug concentrations may better reflect the time to maximum exposure, especially for those compound/preparations, which are typically used without dose escalation, with plasma concentrations slowly rising to this steady‐state because of slow elimination from the circulation (details not shown). This suggests that details of dose escalation regimens are associated with, and may predict, the development of tolerance for nausea and/or vomiting.

### 3.6 Dose‐Escalation Regimens Are Associated With Therapeutic Efficacy (on HbA_1c_ and Body Weight)

**FIGURE 4:**
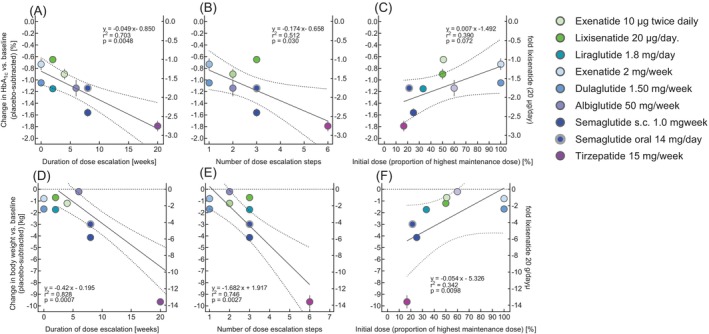
Relationship of characteristics of approved dose‐escalation regimens (duration of dose escalation, number of dose escalation steps and the initial dose as a proportion of the highest maintenance dose) for GLP‐1 receptor agonists and the dual GIP/GLP‐1 receptor agonist tirzepatide to pooled effect sizes for reducing HbA_1c_ and body weight with the highest approved doses of exenatide b.i.d., lixisenatide, liraglutide, exenatide q.w., dulaglutide, albiglutide, semaglutide s.c., semaglutide p.o. and tirzepatide. Effect sizes: Mean values ± standard errors of the mean [22]. Right *x*‐axes show the fold difference to the least effective incretin mimetic, lixisenatide, to display the range of efficacy represented between various incretin mimetics studied. Statistical analysis: Linear regression analysis, reporting the regression equation, *r* ^2^ and the related *p* value. For details of characteristics (duration of dose escalation, number of dose escalation steps and the initial dose as a proportion of the highest maintenance doses) of approved dose‐escalation regimens, see Table 1.

As depicted in Figure 4, linear regression analysis showed significant associations between the duration of dose escalation periods (Figure 4A,D), the number of dose‐escalation steps (Figure 4B,E), and initial doses expressed as a proportion (percentage) of the intended highest maintenance dose (after dose escalation) (Figure 4C,F) and the efficacy of intended therapeutic actions (reductions in HbA_1c_ and body weight). Similar results were found for the reduction in fasting plasma glucose (details not shown). These results indicate that characteristics of dose‐escalation regimens may even be related to and predict therapeutic effect sizes. In line with these results, the ED_50_ ratios estimated from dose–response relationships for nausea and vomiting in Phase 3 versus Phase 1 studies were also related to the efficacies of the compounds/preparations represented in this analysis (reductions in HbA_1c_ and body weight; Figure S4).

## 4 Discussion

The key findings from the current analysis were that dose‐escalation regimens lasting longer (up to 20 weeks) and employing multiple (up to 6) dose steps may contribute to the development of tolerance for nausea and vomiting. This may, in particular, apply to the most elaborate dose‐escalation regimens that have been empirically established for semaglutide and tirzepatide (Figures 1 and 2). Slowly increasing exposure to incretin mimetics may trigger such a process. This supports our empirical observation and hypothesis that appropriately designed dose‐escalation regimens may enable administration of higher doses, rendered tolerable through the induction of tolerance, which in turn may explain greater efficacy for intended clinical outcomes, including but not limited to HbA_1c_ and body weight. While tolerability and efficacy have traditionally been attributed to the specific compounds/preparations within the class of incretin mimetics (GLP‐1 receptor agonists and the GIP/GLP‐1 dual receptor agonist tirzepatide), our results suggest that both may also vary with the use of more or less appropriate dose‐escalation regimens—characterised by a longer duration and multiple dose steps—rather than from the compound itself. The evolution of dose‐escalation regimens (Table 1, Figure S1) indicates empirical learning in this direction. To our knowledge, a systematic analysis of the potential influence of the details of dose‐escalation regimens on the development of tolerance for nausea and vomiting has not been undertaken previously.

In our analyses, we introduce a novel parameter to quantitatively assess the development of gastrointestinal tolerance, derived from dose–response relationships for the proportion of exposed participants reporting nausea or vomiting. As a standardised measure, we estimated the dose (daily or weekly), expected to elicit nausea or vomiting in 50% of study participants (ED_50_). The ED_50_ values were substantially higher than the doses used in Phase 3 clinical trials and subsequently approved, and were higher for most compounds/preparations with respect to eliciting vomiting as compared to nausea (Table S4). For semaglutide (for subcutaneous or oral administration) and tirzepatide, these ED_50_ values were considerably higher when derived from tolerability data obtained from Phase 3 versus Phase 1 clinical studies (Table S4), such that the ratio (ED_50_ from Phase 3 over Phase 1; Figure 1C,D) was significantly higher than 1, indicating that after going through the recommended dose‐escalation regimens, 2–5‐fold higher doses of semaglutide and tirzepatide can be tolerated. This was also consistently observed from the dose–response relationships across the whole dose‐range studied across various agents (Figures 1 and 2).

It should be assumed that, as tolerance develops, the relationship between exposure (circulating plasma concentrations) and the risk of developing nausea and/or vomiting may change over time. Population pharmacokinetic data linking plasma concentrations of GLP‐1 receptor agonists to the dose‐escalation time course are provided in Table S5.

In our prior analysis of the same placebo‐controlled trials of incretin mimetics used for the treatment of Type 2 diabetes [22], one prominent finding was that the risk for gastrointestinal adverse events (expressed as the odds ratio vs. placebo treatment of the proportions of subjects reporting nausea or vomiting) was similar across all compounds (at their highest approved doses), indicating that the doses for Phase 3 studies and for approval were ‘gauged’ to an acceptable level of adverse events at the population level.

A recent clinical study further suggests that extending the dose‐escalation, using even smaller than the established dose steps, may be a feasible way to mitigate gastrointestinal adverse events further [26]. While there are practical limits to extending the standard dose‐escalation period substantially, such tailoring may still be beneficial for selected patients.

Similar analyses could be considered for other tolerability endpoints, such as the discontinuation of randomised treatment, overall or due to adverse events. However, comparisons of discontinuation rates between short, early‐phase studies (with limited opportunity for tolerance to develop) and longer, later‐phase trials (which include escalation periods that facilitate tolerance) are likely to be confounded by these fundamental differences in study design and context.

The magnitude of tolerance development was significantly associated (by linear regression analysis) with the duration of the respective dose escalation period and the number of dose steps (Figure 3). The observed relationship should be considered exploratory, since features of the dose‐escalation regimens may have been chosen in response to tolerability signals during clinical development, leaving a risk of common‐cause confounding.

Furthermore, this ratio also was associated with each agent's efficacy for intended clinical effects (reductions in HbA_1c_ and body weight; Figure S4). Taken together, key parameters characterising the dose‐escalation regimens themselves were associated (by linear regression analysis) with each agent's efficacy for intended clinical efficacy (Figure 4). Given that the efficacy of incretin mimetics captured in head‐to‐head comparisons and network meta‐analyses can materially differ, the between‐drug regression may be prone to being confounded. Therefore, these associations remain hypothesis‐generating and warrant more direct comparative studies in the future.

Because dose selection for Phase 3 clinical trials is determined by the balance of efficacy and tolerability, induction of tolerance through appropriate dose‐escalation regimens may allow the use of higher dose ranges for Phase 3 programmes, potentially by up to a five‐fold difference (Figure 1).

In principle, attenuation of ‘gastrointestinal’ adverse events had already been described in clinical trials of the first GLP‐1 receptor agonist approved for the treatment of Type 2 diabetes, exenatide twice daily [21]; however, treatment was initiated at 50% of the intended maintenance dose, with only a relatively short period (4 weeks) at the initial dose. Along the same lines, modifications of dose‐escalation regimens as suggested by our analysis have been shown to reduce nausea and vomiting in several Phase 2 trials [23, 27, 28]. Moreover, studies with fixed‐dose combinations of a GLP‐1 receptor agonist (e.g., liraglutide or lixisenatide) and a basal insulin, which require much more slow titration due to the insulin component and involve a greater number of dose steps, further confirm that this ‘start low, go slow’‐approach is an excellent way to avoid ‘gastrointestinal’ adverse events both immediately after starting treatment and long‐term, thereby allowing to maximise benefits from incretin mimetics treatment [29, 30, 31, 32].

Insights into tolerance development for nausea and vomiting with incretin mimetics has enabled the subsequent introduction of higher doses than those studied in the original Phase 3 trials. This has been observed for liraglutide (3.0 mg for obesity [33, 34]), dulaglutide (3 and 4.5 mg weekly for Type 2 diabetes [35, 36]), s.c. semaglutide (2.0 mg once weekly [37] for Type 2 diabetes; 2.4 mg once weekly for obesity/overweight [38], 16 mg/week for obesity/overweight and Type 2 diabetes [39]) and oral semaglutide (25 and 50 mg per day [40] for Type 2 diabetes and 50 mg for obesity/overweight [41]). As higher doses are typically required for the treatment of obesity/overweight, induction of tolerance may be even of greater importance for this indication.

Such successful dose‐escalation regimens can serve as a template for the future development of incretin mimetics and related agents with similar adverse event profiles (e.g., dual glucagon/GLP‐1 or GIP/GLP‐1 receptor agonists [42, 43], triple GIP/GLP‐1/glucagon receptor agonists [44, 45]) and agents like maridebart cafraglutide (a GIP receptor‐antagonistic antibody coupled to a GLP‐1 receptor agonist [46]): A key determinant of a successful clinical development appears to be the selection of an optimised initial dose that is low enough to avoid a substantial burden of vomiting or drug discontinuation. Whether a certain degree of nausea is required to initiate the process(es) underlying the development of tolerance is currently unknown. However, indirect evidence comes from a recent study of maridebart cafraglutide, in which nausea and vomiting were reported in nearly all participants, but mainly during the first 2 weeks of treatment, with the prevalence then declining to negligible values. This suggests the possibility that the initial adverse event burden may contribute to triggering the process of developing tolerance for nausea and vomiting. Further research is needed to elucidate the mechanisms underlying these adverse events, including, but not limited, to how it is triggered within the CNS (e.g., the *area postrema* [47]), and to delineate the conditions that promote the development of tolerance.

Once the initial dose has been defined, current evidence supports a dose‐escalation regimen designed to reach the full dose after approximately 20 weeks, using multiple dose steps (up to six) at intervals of approximately 4 weeks. Adhering to this approach may help minimise adverse events during the early phase of future drug development. Often, even in Phase 1 studies, disproportionate emphasis is placed on achieving large effects for improving glycaemic parameters or lowering body weight. This almost inevitably comes at the expense of avoidable adverse events.

Our study has limitations. We used published data from clinical trials with heterogeneous populations, and various study designs including number of participants, study durations and background glucose‐lowering medications. This is particularly relevant for Phase 1 and Phase 2 studies we used in this study to derive an index for the development of tolerance to nausea and vomiting. Particularly, the Phase 1 programmes differ largely in recruitment strategies (e.g., healthy participants versus those with Type 2 diabetes) as well as in the use of single versus multiple doses, immediate exposure versus dose escalation, and the reporting of relevant adverse events (in some cases, only side effects occurring in at least 5% or 10% of any experimental group were reported). In addition, the capture and documentation of nausea and vomiting in the study files of the original studies used for this meta‐analysis was not homogenous or standardised. These are limitations inherent to the methodology of our study, as it had to rely on those data that were publicly available. Also, the major conclusions are based on correlations, so that we cannot claim to have proven causality. On the other hand, the major strengths of the present study are the overall patient numbers, and the use of placebo‐controlled studies, which enabled a reliable comparison of efficacy and tolerability across various compounds/preparations that fall within the incretin mimetic class.

In conclusion, based on a thorough analysis of dose–response relationships for nausea or vomiting across Phase 1 through Phases 3 and 4 clinical trials, we present evidence that appropriate dose‐escalation regimens for the initiation of a therapy with GLP‐1 receptor agonists and the GIP/GLP‐1 dual receptor agonist tirzepatide in patients with Type 2 diabetes may be related to better tolerance for nausea and vomiting. Key parameters characterising these optimal regimens such as the duration of the dose‐escalation period, and the number of dose steps appear to be associated with the development of tolerance, which in turn correlated with the efficacy related to HbA_1c_ and body weight reduction. Prospective studies should confirm whether better tolerance profile achieved through these dose titration strategies enables the exposure to higher doses in Phase 3 clinical trials, thereby leading to greater efficacy. Applying these insights to future drug‐development programmes may help reduce the burden of adverse events associated with incretin mimetics, particularly during early‐phase clinical development.

## Author Contributions

Design of the study (including registration of the protocol with PROSPERO): M.A.N., V.P. and S.L. Literature search: M.A.N., V.P. and Y.M.K. Data analysis: M.A.N., V.P., Y.M.K. and S.L. Drafting of figures and tables: M.A.N., V.P., Y.M.K. and S.L. First manuscript draft: M.A.N. Revision of the manuscript for critical intellectual content and decision to submit the manuscript for publication: M.A.N., V.P., Y.M.K. and S.L. M.A.N. is the guarantor who takes full responsibility for the work. All authors agreed to submit the manuscript for publication.

## Funding

The authors have nothing to report.

## Conflicts of Interest

M.A.N. has been a member on advisory boards or has consulted with Eli Lilly & Co., Pfizer, Regor, Sun Pharma and Structure Therapeutics (Gasherbrum). He has also served on the speakers' bureau of Eli Lilly & Co., Medscape, Medical Learning Institute and NovoNordisk. V.P. and Y.M.K. declare no conflicts of interest. S.L. received research grants from Merck Sharp & Dohme, Novo Nordisk and LG Chem; and honoraria as a consultant or speaker for AstraZeneca, Boehringer Ingelheim, Abbott, LG Chem, Daewoong Pharmaceutical, Chong Kun Dang Pharmaceutical and Novo Nordisk.

## Supporting information


**Table S1:** Clinical trials studying clinical effects of GLP‐1 receptor agonists and the dual GIP/GLP‐1 co‐agonist tirzepatide compared to placebo treatment in subjects with Type 2 diabetes and providing data for the present systematic analysis.
**Table S2:** Studies analysed for examining the development of tolerance to the tolerability endpoints nausea and vomiting between studies at various development stages (Phase 1: immediate exposure, no dose escalation; Phase 2: short dose‐escalations periods; Phase 3: Optimised, longer dose escalation periods, as recommended for the initiation of treatment in clinical practice).
**Figure S1:** Patient numbers, study duration and duration of dose‐escalation periods in Phase 2 and Phase 3, placebo‐controlled, clinical trials with incretin mimetics (GLP‐1 receptor agonists and the GIP/GLP‐1 receptor co‐agonist tirzepatide). Means ± SEM (only panels B and C). Standard errors of the mean are smaller than the diameter of the symbols, and, therefore, often not visible. Phase 3 results were taken from a systematic pooled analysis.
**Table S3:** Number of subjects reporting nausea and vomiting in the studies contributing to estimating ED50ies in development Phases 1, 2 and 3 with approved incretin mimetics (GLP‐1 receptor agonists and the GIP/GLP‐1 receptor dual agonist tirzepatide).
**Table S4:** Estimated ED50ies (non‐linear regression analysis, curve‐fitting) from proportions of study participants reporting nausea or vomiting with each tested dose of incretin mimetic medications (GLP‐1 receptor agonists and the GIP/GLP‐1 receptor dual agonist tirzepatide) by clinical development Phases 1 to 3/4.
**Table S5:** Time course of plasma drug concentrations during the recommended dose‐escalation regimens for GLP‐1 receptor agonists and the GIP/GLP‐1 dual receptor agonist tirzepatide based on published population pharmacokinetic data.
**Figure S2:** Lack of evidence for the development of tolerance against nausea and vomiting with incretin mimetics developed earlier (years 2006–2009: GLP‐1 receptor agonists exenatide twice daily, lixisenatide and liraglutide) based on a comparison of dose‐ effect relationships reported from Phases 1, 2 and 3 clinical trials. Final doses (attained after dose‐escalation) of incretin mimetics (x‐axis) are plotted against the reported proportions of subjects reporting nausea (left hand panels, A, C, E) and vomiting (right hand panels, B, D, F) in studies attributed to Phase 1 (blue; healthy: open symbols; Type 2‐diabetic patients: filled symbols), Phase 2 (green), or Phase 3 (black) with exenatide b.i.d. (A, C), lixisenatide (C, D) and liraglutide (E, F). Dose response‐relationships and their 95% confidence bands derived from curve‐fitting are shown as dotted lines in the same colours. Table S4 lists the effective doses projected to elicit nausea and vomiting in 50% of the subjects and their 95% confidence intervals.
**Figure S3:** Lack of evidence for the development of tolerance against nausea and vomiting with incretin mimetics commonly used without dose escalation (GLP‐1 receptor agonists exenatide once weekly, dulaglutide once weekly and albiglutide once weekly) based on a comparison of dose‐ effect relationships reported from Phases 1, 2 and 3 clinical trials. Final doses (attained after dose‐escalation) of incretin mimetics (x‐axis) are plotted against the reported proportions of subjects reporting nausea (left hand panels, A, C, E) and vomiting (right hand panels, B, D, F) in studies attributed to Phase 1 (blue symbols; healthy subjects: open symbols; Type 2‐diabetic patients: filled symbols), Phase 2 (green), or Phase 3 (black) with exenatide once weekly (A, B), dulaglutide (C, D) and albiglutide (E, F). Dose response‐relationships and their 95% confidence intervals derived from curve‐fitting are shown as dotted lines in the same colours. Table S4 lists the effective doses projected to elicit nausea and vomiting in 50% of the subjects and their 95% confidence intervals.

## Data Availability

The analysis in our manuscript entirely relies on published material. nevertheless, we would be willing to share the data files generated by the collection of published data. Please approach the corresponding author.
