# Supplementary material for: Dose‐Escalation Regimens for Incretin Mimetics in Type 2 Diabetes Are Associated With Tolerance for Nausea and Vomiting
Source: Diabetes Obes Metab. 2026 Feb 27;28(5):4232–42. doi: 10.1111/dom.70613 (PMC13071187; doi:10.1111/dom.70613)
Supplement: Supplementary file 1 — Table S1: Clinical trials studying clinical effects of GLP‐1 receptor agonists and the dual GIP/GLP‐1 co‐agonist tirzepatide compared to placebo treatment in subjects with Type 2 diabetes and providing data for the present systematic analysis. Table S2: Studies analysed for examining the development of tolerance to the tolerability endpoints nausea and vomiting between studies at various development stages (Phase 1: immediate exposure, no dose escalation; Phase 2: short dose‐escalations periods; Phase 3: Optimised, longer dose escalation periods, as recommended for the initiation of treatment in clinical practice). Figure S1: Patient numbers, study duration and duration of dose‐escalation periods in Phase 2 and Phase 3, placebo‐controlled, clinical trials with incretin mimetics (GLP‐1 receptor agonists and the GIP/GLP‐1 receptor co‐agonist tirzepatide). Means ± SEM (only panels B and C). Standard errors of the mean are smaller than the diameter of the symbols, and, therefore, often not visible. Phase 3 results were taken from a systematic pooled analysis. Table S3: Number of subjects reporting nausea and vomiting in the studies contributing to estimating ED50ies in development Phases 1, 2 and 3 with approved incretin mimetics (GLP‐1 receptor agonists and the GIP/GLP‐1 receptor dual agonist tirzepatide). Table S4: Estimated ED50ies (non‐linear regression analysis, curve‐fitting) from proportions of study participants reporting nausea or vomiting with each tested dose of incretin mimetic medications (GLP‐1 receptor agonists and the GIP/GLP‐1 receptor dual agonist tirzepatide) by clinical development Phases 1 to 3/4. Table S5: Time course of plasma drug concentrations during the recommended dose‐escalation regimens for GLP‐1 receptor agonists and the GIP/GLP‐1 dual receptor agonist tirzepatide based on published population pharmacokinetic data. Figure S2: Lack of evidence for the development of tolerance against nausea and vomiting with incretin mimetics developed earl [file DOM-28-4232-s001.docx]

**Supplementary Tables and Figures**

**Dose-escalation regimens for incretin mimetics in type 2 diabetes are associated with tolerance for nausea and vomiting**

Michael A. Nauck^1^, Viktoria Punov^1^, Yu Mi Kang^2^, and Soo Lim^3^

^1^Diabetes, Endocrinology, Metabolism Section Medical Department I. Josef-Hospital, Ruhr University Bochum, Bochum, Germany, michael.nauck@rub.de

^2^Division of Endocrinology, Diabetes and Hypertension and TIMI Study Group, Brigham and Women's Hospital, Harvard Medical School, Boston, MA, USA, ymkang@bwh.harvard.edu

^3^Seoul National University College of Medicine, Seoul National University Bundang Hospital, Seongnam-city, South Korea, limsoo@snu.ac.kr

| **Supplementary Table 1.** Clinical trials studying clinical effects of GLP-1 receptor agonists and the dual GIP/GLP-1 co-agonist tirzepatide compared to placebo treatment in subjects with type 2 diabetes and providing data for the present systematic analysis | | | | | |
| --- | --- | --- | --- | --- | --- |
| Study number | Compound | Publication | Dosage | Study Acronym | Duration (weeks) |
| 1 | Exenatide b.i.d. | Buse et al. 2004 [1] | 5 or 10 µg twice daily | AMIGO 1 | 30 |
| 2 |  | DeFronzo et al. 2005 [2] | 5 or 10 µg twice daily | AMIGO 2 | 30 |
| 3 |  | Kendall et al. 2005 [3] | 5 or 10 µg twice daily | AMIGO 3 | 30 |
| 4 | Lixisenatide | Fonseca et al. 2012 | 20 µg once daily | GetGoal Mono | 12 |
| 5 |  | Ahren et al. 2013 [4] | 20 µg once daily | GetGoal-M | 24 |
| 6 |  | Pinget et al. 2013 [5] | 20 µg once daily | GetGoal-P | 24 |
| 7 |  | Seino et al. 2012 [6] | 20 µg once daily | GetGoal -L-Asia | 24 |
| 8 |  | Riddle et al. 2013 [7] | 20 µg once daily | GetGoal-L | 24 |
| 9 |  | Bolli et al. 2014 [8] | 20 µg once daily | GetGoal-F 1 | 24 |
| 10 |  | Yu Pan et al. 2014 [9] | 20 µg once daily | GetGoal-M-Asia | 24 |
| 11 |  | Rosenstock et al. 2014 [10] | 20 µg once daily | GetGoal-S | 24 |
| 12 | Liraglutide | Marre et al. 2009 [11] | 0.6, 1.2, or 1.8 mg per day | LEAD 1 | 26 |
| 13 |  | Nauck et al. 2009 [12] | 0.6, 1.2, or 1.8 mg per day. | LEAD 2 | 26 |
| 14 |  | Bernard Zinman [13] | 1.2, or 1.8 mg per day | LEAD 4 | 26 |
| 15 |  | Russell-Jones et al. 2009 [14] | 1.8 mg per day | LEAD 5 | 26 |
| 16 |  | Blonde et al. 2020 [15] | 1.8 mg per day | LIRA ADD2SGLT2 | 26 |
| 17 | Exenatide q.w. | Gadde et al. 2017 [16] | 2 mg per week | DURATION NEO 2 | 28 |
| 18 |  | Guja et al. 2018 [17] | 2 mg per week | DURATION 7 | 28 |
| 19 | Dulaglutide | Wysham et al. 2014 [18] | 0.75 or 1.5 mg per week | AWARD 1 | 26 |
| 20 |  | Skrivanek et al. 2014 [19] | 0.75 or 1.5 mg per week | AWARD 5 | 26 |
| 21 |  | Dungan et al. 2016 [20] | 1.5 mg per week | AWARD 8 | 24 |
| 22 |  | Pozzilli et al. 2017 [21] | 1.5 mg per week | AWARD 9 | 28 |
| 23 |  | Ludvik et al. 2018 [22] | 0.75 or 1.5 mg per week | AWARD 10 | 24 |
| 24 | Albiglutide | Reusch et al. 2014 [23] | 30 mg per week | HARMONY 1 | 52 |
| 25 |  | Nauck et al. 2016 [24] | 30 or 50 mg per week | HARMONY 2 | 52 |
| 26 |  | Åhren et al. 2014 [25] | 30 mg per week | HARMONY 3 | 104 |
| 27 |  | Home et al. 2015 [26] | 30 mg per week | HARMONY 5 | 52 |
| 28 | Semaglutide s.c. | Sorli et al. 2017 [27] | 0, 5 or 1 mg per week | SUSTAIN 1 | 30 |
| 29 |  | Rodbard et al. 2018 [28] | 0, 5 or 1 mg per week | SUSTAIN 5 | 30 |
| 30 |  | Zinman et al. 2019 | 1 mg per week | SUSTAIN-9 |  |
| 31 | Semaglutide oral | Aroda et al. 2019 [29] | 3, 7, or 14 mg per day | PIONEER 1 | 26 |
| 32 |  | Pratley et al. 2019 [30] | 14 mg per day | PIONEER 4 | 52 |
| 33 |  | Mosenzon et al. 2019 [31] | 14 mg per day | PIONEER 5 | 26 |
| 34 |  | Zinman et al. 2019 [32] | 3, 7, or 14 mg per day | PIONEER 8 | 26 |
| 35 |  | Yamada et al. 2020 [33] | 3, 7, or 14 mg per day | PIONEER 9 | 52 |
| 36 | Tirzepatide | Rosenstock et al. 2021 [34] | 5, 10, or 15 mg per week | SURPASS-1 | 40 |
| 37 |  | Dahl et al. 2022 [35] | 5, 10, or 15 mg per week | SURPASS-5 | 40 |
| Studies were selected from the pivotal clinical trial programs supporting approval of the respective GLP-1 receptor agonist or dual GIP/GLP-1 receptor co-agonist tirzepatide (identified by the typical acronym for each development program). For details regarding the search strategy, see Supplementary Figure 1 (PRISMA flow diagram). Only studies providing placebo-subtracted effect sizes for the main outcome parameters HbA_1c_, fasting plasma glucose concentration, and body weight reduction were selected. | | | | | |

**Supplementary Figure 1. Patient numbers, study duration, and duration of dose-escalation periods in phase 2 and phase 3, placebo-controlled, clinical trials with incretin mimetics (GLP-1 receptor agonists and the GIP/GLP-1 receptor co-agonist tirzepatide).** Means ± SEM (only panels B and C). Standard errors of the mean are smaller than the diameter of the symbols, and, therefore, often not visible. Phase 3 results were taken from a systematic pooled analysis .

| **Supplementary Table 2.** Studies analysed for examining the development of tolerance to the tolerability endpoints nausea and vomiting between studies at various development stages (phase 1: immediate exposure, no dose escalation; phase 2: short dose-escalations periods; phase 3: Optimized, longer dose escalation periods, as recommended for the initiation of treatment in clinical practice) | | | | | | | |
| --- | --- | --- | --- | --- | --- | --- | --- |
| Incretin mimetic compound/ preparation | Clinical trial phase | Subjects | Source (first author, year) | Dosing | Dose range | Duration | Commentary |
| Exenatide b.i.d. | Phase 1 | Healthy subjects | Linnebjerg et al. 2007 [36] | Single s.c. dose | 10 µg | 1 day | Only results from subjects with normal renal function analysed; no placebo control reported |
|  |  | T2DM | Kolterman et al. 2005 [37] | Ascending or randomized single s.c. doses | 0.01-0.4 µg/ kg body weight and placebo | 3-9 days | The doses correspond to 0.9 - 35.4 µg |
|  | Phase 2 | T2DM | Fineman et al. 2003 [38] | 2-3 times daily before meals | 0.08 µg and placebo | 28 days | The dose corresponds to 7.8 µg (1-3 times daily) |
| Lixisenatide | Phase 2 | T2DM | Ratner et al. 2010 [39] | One s.c. dose daily (before breakfast) | 5-30 µg and placebo | 13 weeks | Only single injections per day analysed (results for twice daily injections also reported) |
| Liraglutide | Phase 1 | Healthy subjects | Agersø et al. 2002 [40] | Once daily s.c. | 1.25 to 12.5 µg/kg and placebo | Single dose and 11 days | The doses correspond to 1.0 – 10.1 mg |
|  |  |  | Flint et al. 2010 [41] | Single s.c. dose | 0.75 mg | 1 day | Only results from subjects with normal hepatic function analysed; no placebo control reported |
|  |  |  | Jacobsen et al. 2009 [42] | Single s.c. dose | 0.75 mg | 1 day | Only results from subjects with normal renal function analysed; no placebo control reported |
|  | Phase 2 | T2DM | Madsbad et al. 2004 [43] | Once daily s.c | 0.045, 0.225, 0.45, 0.60, 0.75 mg/day | 12 weeks | The exposure to low (and largely ineffective) doses made a second wave of phase 2 studies necessary (see Nauck et al. 2006 [44]) |
|  |  |  | Feinglos et al. 2005 [45] | Once daily s.c | 0.045, 0.225, 0.45, 0.60, 0.75 mg/day | 2 weeks | The exposure to low (and largely ineffective) doses made a second wave of phase 2 studies necessary (see Nauck et al. 2006 [44]) |
|  |  |  | Nauck et al. 2006 [44] | 0.5, 1.0, 1.5, and 2.0 mg s.c. daily for one week each | Final dose: 2.0 mg per day | 5 weeks | Only data on metformin co-treatment analysed |
| Exenatide q.w. | Phase 1 | T2DM | Fineman et al. 2011 [46] | Single s.c. dose | 0-10 mg and placebo | 1 day | Vomiting was not reported with exact numbers, (only events with > 10 % incidence were explicitly reported) |
|  | Phase 2 | T2DM | Kim et al. 2007 [47] | Weekly s.c. injections | 0.8 and 2.0 mg weekly and placebo | 15 weeks | No vomiting was stated in the text |
|  |  |  | Cui et al. 2013 [48] | Weekly s.c. injections | 0, 0.8 and 2.0 mg weekly | 10 weeks (with another 10 weeks of observation) | Chinese patients; no control group |
|  |  |  | Iwamoto et al. 2009 [49] | Weekly s.c. injections | 0.8 and 2.0 mg weekly and placebo | 8 weeks | Japanese patients |
| Table continued on next page | | | | | | | |
| Table continued from previous page | | | | | | | |
| Incretin mimetic compound/ preparation | Clinical trial phase | Subjects | Source (first author, year) | Dosing | Dose range | Duration | Commentary |
| Dulaglutide | Phase 1 | T2DM | Barrington et al. 2011 [50] | Weekly s.c. injections | 0.05 to 8.0 mg and placebo | 5 weeks | Adverse events only presented as text |
| Dulaglutide (continued) | Phase 1 (continued) |  | Umpierrez et al. 2011 [51] | Weekly s.c. injections | 0.5 mg, then 1.0 mg; 1.0 mg; 1.0 mg, then 2.0 mg | 16 weeks (dose escalation after 4 weeks) | No placebo control studied |
|  |  |  | Xu et al. 2022 [52] | Weekly s.c. injections | 0,5, 0.75, and 1.5 mg and placebo | 4 weeks (29 days) | Chinese patients, single dose study without placebo control |
|  | Phase 2 | T2DM | Grunberger et al. 2012 [53] | Weekly s.c. injections | 0.5, 1.0, 1.5 mg and placebo | 12 weeks | Safety data presented as text |
|  |  |  | Terauchi et al. 2014 [54] | Weekly s.c. injections | 0.25, 0.5, or 0.75 mg | 12 weeks | Japanese patients; vomiting not exactly reported (only events with an incidence ≥ 5 % in any treatment group reported) |
|  |  |  | Skrivanek et al. 2014 [19] | Weekly s.c. injections | 0.25 to 3.0 mg | 20-30 weeks | Patients were followed until treatment arms were discontinued due to an unfavourable “clinical utility index” (adaptive, seamless design) |
| Albiglutide | Phase 1 | Healthy | Shaddinger et al. 2019 [55] | Single s.c. injections | 50 mg | 1 day | Only results obtained with re-dissolved lyophilized albiglutide were analysed |
|  |  | T2DM | Matthews et al. 2008 [56] | Weekly s.c. injections | 9, 16, 32, or 64 mg | 1 or 2 weeks | 16 and 64 mg also tested with s.c. injections into the arm, leg, or abdomen |
|  |  | Healthy | Young et al. 2014 [57] | Single s.c. injections | 30 mg | 1 day | Only results from subjects with normal renal function analysed |
|  | Phase 2 | T2DM | Rosenstock et al. 2009 [58] | Weekly s.c. injections | Placebo, 4, 15, and 30 mg | 16 weeks | Only once-weekly injections were analysed (biweekly and monthly injections were disregarded) |
|  |  |  | Seino et al. [59] | Weekly s.c. injections | Placebo, 15, or 30 mg | 16 weeks | Japanese patients¸ Only once-weekly injections were analysed (biweekly injections were disregarded); a study in patients with T2DM and renal impairment were not analysed |
| Semaglutide s.c. | Phase 1 | Healthy | Marbury et al. 2017 [60] | Single s.c. injection | 0.5 mg | 1 day | Only results from subjects with normal renal function analysed |
|  |  |  | Jensen et al. 2018 [61] | Single s.c. injection | 0.5 mg | 1 day | Only results from subjects with normal hepatic function analysed |
|  |  | T2DM | Kapitza et al. 2015 [62] | Weekly s.c. injections | Sequentially 0.25, 0.5, and 1.0 m | 13 weeks (dose escalation after 4 weeks) | Study performed to determined absorption of estrogen/progesterone |
|  | Phase 2 | T2DM | Nauck et al. 2016 [63] | Weekly s.c. injections | Placebo, 0.1 to 1.6 mg (final doses) | 12 weeks (dose escalation for doses ≥ 0.8 mg) | - |
| Table continued on next page | | | | | | | |
| Table continued from previous page | | | | | | | |
| Incretin mimetic compound/ preparation | Clinical trial phase | Subjects | Source (first author, year) | Dosing | Dose range | Duration | Commentary |
| Semaglutide oral | Phase 1 | Healthy | Bækdal et al. 2018 [64] | Daily oral administration | 3 and 7 mg for 5 days each | 10 days | Only results from subjects with normal hepatic function analysed; nausea not exactly reported |
|  |  | Healthy | Granhall et al. 2019 [65] | Daily oral administration | Placebo, 20 and 40 mg | 10 weeks | Results with placebo and SNAC (the relevant absorption enhancer) were analysed (SNAC was also co-administered with oral semaglutide) |
|  |  | Healthy | Van Hout et al. 2023 [66] | Daily oral administration | 3 and 7 mg for 5 days each | 10 days | Only results with overnight pre-dose fast and 30 min post-dose fast were analysed (identical to recommendations for approved doses) |
|  | Phase 2 | T2DM | Davies et al. 2017 [67] | Daily oral administration | 2.5 to 40 mg daily (dose escalation for doses > 2.5 mg) | 26 weeks | The final dose 40 mg per day was achieved with dose escalation periods lasting from 6 to 12 and 24 weeks, which slightly modified the incidence of nausea |
| Tirzepatide | Phase 1 | Healthy | Coskun et al. 2018 [68] | Single s.c. injection | 0.25, 0.5, 1.0, 2.5, 5.0, and 8.0 mg and placebo | 1 day | Tirzepatide as introduced as LY3298176 in this manuscript |
|  |  | T2DM | Coskun et al. 2018 [68] | Weekly s.c. injections | 0.5, 5, 10, and 15 mg (rapid dose escalation for 10 and 15 mg) and placebo | 29 days | Tirzepatide as introduced as LY3298176 in this manuscript |
|  |  | T2DM | Furihata et al. 2022 [69] | Weekly s.c. injections | 5, 10, and 15 mg (rapid dose escalation for 10 and 15 mg doses) | 8 weeks | Japanese subjects |
|  | Phase 2 | T2DM | Frias et al. 2018[70] | Weekly s.c. injections | Placebo, 1 to 15 mg (rapid dose escalation over 2-6 weeks for 10 and 15 mg doses) | 26 weeks | Tirzepatide as introduced as LY3298176 in this manuscript |
|  |  | T2DM | Frias et al. 2020 [70] | Weekly s.c. injections | Placebo, 12, 15 mg (rapid dose escalation) | 12 weeks | Two different dose escalation regimens were used for the 15 mg per week dose; no placebo control. |
| T2DM: Type 2 diabetes mellitus | | | | | | | |

| **Supplementary Table 3.** Number of subjects reporting nausea and vomiting in the studies contributing to estimating ED50ies in development phases 1, 2 and 3 with approved incretin mimetics (GLP-1 receptor agonists and the GIP/GLP-1 receptor dual agonist tirzepatide) | | | | | | | | | | | | |
| --- | --- | --- | --- | --- | --- | --- | --- | --- | --- | --- | --- | --- |
|  |  | Phase 1 |  |  |  | Phase 2 |  |  |  | Phase 3 |  |  |
| A. Nausea |  | N reporting nausea | N total | Proportion reporting nausea [%] |  | N reporting nausea | N total | Proportion reporting nausea [%] |  | N reporting nausea | N total | Proportion reporting nausea [%] |
| Exenatide b.i.d. |  | 10 | 85 | 11.8 |  | 16 | 81 | 19.8 |  | 505 | 1446 | 34.9 |
| Lixisenatide |  | n.r. | n.r. | n.r. |  | 48 | 325 | 14.8 |  | 720 | 4062 | 17.8 |
| Liraglutide |  | 6 | 19 | 31.6 |  | 19 | 72 | 26.5 |  | 237 | 2025 | 11.7 |
| Exenatide q.w. |  | 3 | 62 | 4.8 |  | 17 | 99 | 17.2 |  | 37 | 703 | 5.3 |
| Dulaglutide |  | 35 | 202 | 11.6 |  | 52 | 497 | 10.5 |  | 214 | 1722 | 12.4 |
| Albiglutide |  | 17 | 196 | 8.9 |  | 4 | 270 | 1.5 |  | 130 | 1391 | 9.3 |
| Semaglutide s.c. |  | 18 | 76 | 23.7 |  | 147 | 316 | 46.5 |  | 154 | 1085 | 14.2 |
| Semaglutide p.o. |  | 48 | 159 | 30.2 |  | 275 | 561 | 49.0 |  | 283 | 2380 | 11.99 |
| Tirzepatide |  | 6 | 56 | 9.0 |  | 178 | 373 | 20.9 |  | 120 | 955 | 12.6 |
| All incretin mimetics |  | 143 | 855 | 16.7 |  | 756 | 2594 | 29.1 |  | 2400 | 15769 | 15.2 |
| B. Vomiting |  | N reporting vomiting | N total | Proportion reporting vomiting [%] |  | N reporting vomiting | N total | Proportion reporting vomiting [%] |  | N reporting vomiting | N total | Proportion reporting vomiting [%] |
| Exenatide b.i.d. |  | 2 | 32 | 6.3 |  | n.r. | n.r. | n.r. |  | 141 | 1496 | 9.8 |
| Lixisenatide |  | n.r. | n.r. | n.r. |  | 19 | 325 | 5.8 |  | 284 | 4062 | 7.0 |
| Liraglutide |  | 1 | 42 | 1.4 |  | 6 | 72 | 8.3 |  | 69 | 2025 | 3.4 |
| Exenatide q.w. |  | n.r. | n.r. | n.r. |  | 6 | 99 | 6.1 |  | 10 | 703 | 1.4 |
| Dulaglutide |  | 23 | 347 | 17.0 |  | 17 | 352 | 4.8 |  | 75 | 1000 | 7.5 |
| Albiglutide |  | 6 | 191 | 3.1 |  | 3 | 270 | 1.1 |  | 33 | 1090 | 3.0 |
| Semaglutide s.c. |  | 7 | 76 | 9.2 |  | 54 | 316 | 17.1 |  | 248 | 1085 | 22.9 |
| Semaglutide p.o. |  | 42 | 211 | 19.9 |  | 79 | 561 | 14.1 |  | 128 | 2185 | 5.9 |
| Tirzepatide |  | 5 | 56 | 11.0 |  | 50 | 384 | 13.0 |  | 51 | 953 | 5.4 |
| All incretin mimetics |  | 86 | 955 | 9.0 |  | 234 | 2379 | 9.8 |  | 1039 | 14581 | 7.1 |
| n.r.: Not reported; numbers include subjects studied with placebo treatment. In some studies, vomiting was not reported, because only adverse events occurring in ≥ 5 % in any group were explicitly reported. Total patient numbers are smaller than reported in individual patients, because studies not reporting nausea or vomiting were not counted. | | | | | | | | | | | | |

| **Supplementary Table 4.** Estimated ED_50_ies (non-linear regression analysis, curve-fitting) from proportions of study participants reporting nausea or vomiting with each tested dose of incretin mimetic medications (GLP-1 receptor agonists and the GIP/GLP-1 receptor dual agonist tirzepatide) by clinical development phases 1 to 3/4. | | | | | |
| --- | --- | --- | --- | --- | --- |
| Tolerability endpoint | Incretin mimetic compound/preparation | (unit) | Effective dose 50 % (ED_50_) | | |
|  |  |  | Phase 1 studies | Phase 2 studies | Phase 3 studies |
| Nausea | Nausea |  |  |  |  |
|  | Exenatide b.i.d. | µg/dose | 170.8 (70.6; ∞) | 38.9 (18.9;175.9) | 15.1 (12.9; 17.9) ^a^ |
|  | Lixisenatide | µg/dose | n.r. | 78.4 (57.8; 113.4) | 83.3 (75.8; 92.0) |
|  | Liraglutide | mg/day | 21.2 (10.0; 75.7) | 5.9 (3.6; 111.6) | 9.5 (8.3; 10.9) |
|  | Exenatide q.w. | mg/week | 135.9 (53.1; ∞) | 8.3 (4.8; 19.6) | 60.1 (35.1; 189.0) ^b^ |
|  | Dulaglutide | mg/week | 12.4 (8.0; 237.0) | 9.2 (6.7; 13.8) | 8.5 (7.2; 10.2) |
|  | Albiglutide | mg/week | 2809 (403; ∞) | 1233 (519.8; ∞) | 1470 (738; 29925) |
|  | Semaglutide s.c | mg/week | 2.5 (0.5; 6.4) | 0.5 (0.4; 0.7) | 6.4 (5.0; 8.5) ^b^ |
|  | Semaglutide oral | mg/day | 48.9 (31.8; 80.0) | 18.2 (14.2; 23.3) | 115.2 (94.3; 146.3) ^a,b^ |
|  | Tirzepatide | mg/week | 15.6 (10.3; 25.8) | 31.4 (24.5; 41.5) | 78.7 (62.8; 103.1) ^a,b^ |
| Vomiting | Vomiting |  |  |  |  |
|  | Exenatide b.i.d. | µg/dose | 117.0 (32.4;∞) | n.a. | 10.23 (8.98: 11.69) ^a^ |
|  | Lixisenatide | µg/dose | n.r. | 208.4 (144.4; 350.8) | 237.9 (207.7; 277.3) |
|  | Liraglutide | mg/day | 106.1 (33.5; ∞) | 22.0 (11.6; 101.0) | 31.4 (25.9; 39.7) |
|  | Exenatide q.w. | mg/week | n.r. | 20.3 (6.2; ∞) | 140.4 (77.6; 672) |
|  | Dulaglutide | mg/week | 14.8 (11.2; 20.5) | 16.7 (11.1; 30.7) | 11.3 (9.2; 14.3) |
|  | Albiglutide | mg/week | 2683 (611.4; ∞) | 750.0 (466.2; 1805.0) | 1350 (867; 2943) |
|  | Semaglutide s.c | mg/week | 10.5 (4.4; ∞) | 2.7 (2.1; 3.8) | 16.5 (11.5; 27.2) ^b^ |
|  | Semaglutide oral | mg/day | 81.1 (57.0; 124.2) | 200.7 (149.0; 292.3) ^a^ | 401.1 (268.1; 773.0) ^a^ |
|  | Tirzepatide | mg/week | 43.3 (30.9; 65.5) | 52.8 (38.3; 79.1) | 209 (152.5; 324.2) ^a,b^ |
| Data are results of non-linear regression analysis (agonist vs. dose) with calculation of the effective dose 50 %, as graphically displayed in Figure 1. Calculated values and 95 % confidence intervals. n.a.: not available; n.d.: not determinable; ED_50_: Effective dose 50 % (dose eliciting the tolerability endpoint in 50 % of the treated subjects under the conditions shown (clinical trials phase). B.i.d.: *bis in diem*, once daily; q.w.: *quantum* per week; once weekly; n.r. data needed for the calculation not reported; n.d.: not determinable; ^a^: significantly (p < 0.05) different from phase 1 studies;  ^b^: significantly (p < 0.05) different from phase 2 studies; ^c^: significantly (p < 0.05) lower than 1; ^d^: significantly (p < 0.05) greater than 1 (comparison based on 95 % confidence intervals overlapping with comparator). | | | | | |

| **Supplementary Table 5.** Time course of plasma drug concentrations during the recommended dose-escalation regimens for GLP-1 receptor agonists and the GIP/GLP-1 dual receptor agonist tirzepatide based on published population pharmacokinetic data. | | | | | | | | | | | | | | | |
| --- | --- | --- | --- | --- | --- | --- | --- | --- | --- | --- | --- | --- | --- | --- | --- |
| Compound/ preparation | Molecular weight (Dalton) | Unit | Plasma concentrations (population pharmacokinetics) | | | | | | | Time to steady state [weeks] | | Reference | Commentary | |  |
|  |  |  | 1 week | 4 weeks | | 8 weeks | | Steady state | |  |  |  |  |  |  |
| Exenatide b.i.d. | 4186.6^a^ | pmol/l | 2.4-26.3 | | 4.8-52.6 | | 4.8-52.6 | | 4.8-52.6 | | 2.5 | Kolterman et al. 2005 [71] | | Read from figures; original values reported as pg/ml; results interpolated for 5 and 10 µg doses (from 0.05, 0.10, and 0.2 µg/kg doses) assuming dose linearity and 75 kg body weight | |
| Lixisenatide | 4858.6^b^ | pmol/l | 0-8.2 | | 0-8.2 | | 0-15.6 | | 0-15.6 | | 2.5 | Barientos-Pérez et al. 2021 [72] | | Measurements from subjects without anti-drug antibodies used (assuming interference of antibodies in the immunoassay) | |
| Liraglutide | 3751.3 | nmol/l^c^ | 2.4-7.4 | | 9.5-17.5 | | 9.5-17.5 | | 9.5-17.5 | | 3.5 | Carlsson-Petrie et al. 2015 [73] | | Read from a figure showing steady state liraglutide concentrations over a 24 h period for 0.6, 1.2, and 1.8 mg liraglutide injected per day for 7 days. | |
| Exenatide q.w. | 4186.6^a^ | pmol/l | 5 | | 21 | | 56 | | 61 | | ≈ 9 | Li et al. 2015 [74] | | Read from figures (T2D, treatment for > 10 weeks); similar profiles described in Chinese (Cui et al. 2013) [75] and Japanese (Iwamoto et al. 2009 [49]) T2D patients | |
|  |  |  | 5.3 | | 33.4 | | 52.5 | | 59.7 | | ≈ 9 | Fineman et al. 2011 [46] | |  |  |
| Dulaglutide | 59669.8 | pmol/l | 469-788 | | 804-1374 | | 922-1391 | | 922-1391 | | 8 | Geiser et al. 2016 [76] | | Read from a figure representing modelled results. Values originally reported as ng/ml. Approximately confirmed in Chinese (Xu et al. 2022 [52] subjects. | |
| Albiglutide | 72970.4 | nmol/l | 10.5-13.3 | | 19.2-20.7 | | n.r. | | n.r | | 4 | Matthews et a. 2008 [56] | | Read form a figure (which displays only 2 injections 1 week apart; also stated in Blair & Keating 2015 [77]) | |
| Semaglutide s.c. | 4113.6 | nmol/l | 3-4 | | 4-7 | | 10-16 | | 20-35 | | 14 | Overgaard et al. 2019 [78] | | Read from a figure displaying results from clinical trials (in healthy and T2D subjects); Steady-state concentrations confirmed by Carlsson-Petrie et al. 2018 [79]); similar values reported in Caucasian and Japanese healthy subjects (Ikushima et al. 2018 [80]) | |
| Semaglutide oral | 4113.6 | nmol/l | 3.2-5.1 | | 3.2-5.1 | | 7.4-11.9 | | 14.7-28.8 | | 14 | Granhall et al. 2019 [81] | | Read from a figure displaying 24 h concentration profiles after 20 and 40 mg oral semaglutide (with 300 mg SNAC) for 10 weeks. Concentrations for doses of 3, 7, and 14 mg were derived by assuming dose-linearity. | |
| Tirzepatide | 4813.45 | nmol/l | 20.8-41.6 | | 41.6-62.3 | | 72.7-124.6 | | 249-374 | | 24 | Schneck & Urva 2023 [82] | | Read from a figure based on model-predicted profiles of tirzepatide concentrations with doses of 2.5- 15 mg per week tirzepatide. | |
| ^a^: 4186.6 pg/l (ng/ml) = 1 pmol/l (originally reported as pg/ml); ^b^: 4858.6 pg/l = 1 pmol/l (originally reported as pg/ml); ^c^: 0.5 to 1.1 % are non-protein bound; *: Estimated as 5 elimination half-lives) | | | | | | | | | | | | | | | |

**ESM Figure 2. Lack of evidence for the development of tolerance against nausea and vomiting with incretin mimetics developed earlier (years 2006-2009: GLP-1 receptor agonists exenatide twice daily, lixisenatide and liraglutide) based on a comparison of dose- effect relationships reported from phase 1, 2 and 3 clinical trials.** Final doses (attained after dose-escalation) of incretin mimetics (x-axis) are plotted against the reported proportions of subjects reporting nausea (left hand panels, A, C, E) and vomiting (right hand panels, B, D, F) in studies attributed to phase 1 (blue; healthy: open symbols; type 2-diabetic patients: filled symbols), phase 2 (green), or phase 3 black) with exenatide b.i.d. (A, C), lixisenatide (C, D), and liraglutide (E, F). Dose response-relationships and their 95 % confidence bands derived from curve-fitting are shown as dotted lines in the same colours. ESM Table4 lists the effective doses projected to elicit nausea and vomiting in 50 % of the subjects and their 95 % confidence intervals.

**ESM Figure 3. Lack of evidence for the development of tolerance against nausea and vomiting with incretin mimetics commonly used without dose escalation (GLP-1 receptor agonists exenatide once weekly, dulaglutide once weekly, and albiglutide once weekly) based on a comparison of dose- effect relationships reported from phase 1, 2 and 3 clinical trials.** Final doses (attained after dose-escalation) of incretin mimetics (x-axis) are plotted against the reported proportions of subjects reporting nausea (left hand panels, A, C, E) and vomiting (right hand panels, B, D, F) in studies attributed to phase 1 (blue symbols; healthy subjects: open symbols; type 2-diabetic patients: filled symbols), phase 2 (green), or phase 3 (black) with exenatide once weekly (A, B), dulaglutide (C, D) and albiglutide (E, F). Dose response-relationships and their 95 % confidence intervals derived from curve-fitting are shown as dotted lines in the same colours. ESM Table 4 lists the effective doses projected to elicit nausea and vomiting in 50 % of the subjects and their 95 % confidence intervals.

**References quoted in the Supplementary Appendix**

[1] Buse JB, Henry RR, Han J, et al. (2004) Effects of exenatide (exendin-4) on glycemic control over 30 weeks in sulfonylurea-treated patients with type 2 diabetes. Diabetes Care 27: 2628-2635

[2] DeFronzo RA, Ratner RE, Han J, Kim DD, Fineman MS, Baron AD (2005) Effects of exenatide (exendin-4) on glycemic control and weight over 30 weeks in metformin-treated patients with type 2 diabetes. Diabetes Care 28: 1092-1100

[3] Kendall DM, Riddle MC, Rosenstock J, et al. (2005) Effects of exenatide (exendin-4) on glycemic control over 30 weeks in patients with type 2 diabetes treated with metformin and a sulfonylurea. Diabetes Care 28: 1083-1091

[4] Ahren B, Leguizamo Dimas A, Miossec P, Saubadu S, Aronson R (2013) Efficacy and safety of lixisenatide once-daily morning or evening injections in type 2 diabetes inadequately controlled on metformin (GetGoal-M). Diabetes Care 36: 2543-2350. 10.2337/dc12-2006

[5] Pinget M, Goldenberg R, Niemoeller E, Muehlen-Bartmer I, Guo H, Aronson R (2013) Efficacy and safety of lixisenatide once daily versus placebo in type 2 diabetes insufficiently controlled on pioglitazone (GetGoal-P). Diabetes Obes Metab 15(11): 1000-1007. 10.1111/dom.12121

[6] Seino Y, Min KW, Niemoeller E, Takami A, Investigators EG-LAS (2012) Randomized, double-blind, placebo-controlled trial of the once-daily GLP-1 receptor agonist lixisenatide in Asian patients with type 2 diabetes insufficiently controlled on basal insulin with or without a sulfonylurea (GetGoal-L-Asia). Diabetes Obes Metab 14: 910-917. 10.1111/j.1463-1326.2012.01618.x

[7] Riddle MC, Aronson R, Home P, et al. (2013) Adding once-daily lixisenatide for type 2 diabetes inadequately controlled by established basal insulin: a 24-week, randomized, placebo-controlled comparison (GetGoal-L). Diabetes Care 36: 2489-2496. 10.2337/dc12-2454

[8] Bolli GB, Munteanu M, Dotsenko S, et al. (2014) Efficacy and safety of lixisenatide once daily vs. placebo in people with Type 2 diabetes insufficiently controlled on metformin (GetGoal-F1). Diabet Med 31: 176-184. 10.1111/dme.12328

[9] Yu Pan C, Han P, Liu X, et al. (2014) Lixisenatide treatment improves glycaemic control in Asian patients with type 2 diabetes mellitus inadequately controlled on metformin with or without sulfonylurea: a randomized, double-blind, placebo-controlled, 24-week trial (GetGoal-M-Asia). Diabetes Metab Res Rev 30: 726-735. 10.1002/dmrr.2541

[10] Rosenstock J, Hanefeld M, Shamanna P, et al. (2014) Beneficial effects of once-daily lixisenatide on overall and postprandial glycemic levels without significant excess of hypoglycemia in type 2 diabetes inadequately controlled on a sulfonylurea with or without metformin (GetGoal-S). J Diabetes Complications 28(3): 386-392. 10.1016/j.jdiacomp.2014.01.012

[11] Marre M, Shaw J, Brandle M, et al. (2009) Liraglutide, a once-daily human GLP-1 analogue, added to a sulphonylurea over 26 weeks produces greater improvements in glycaemic and weight control compared with adding rosiglitazone or placebo in subjects with Type 2 diabetes (LEAD-1 SU). Diabet Med 26: 268-278

[12] Nauck M, Frid A, Hermansen K, et al. (2009) Efficacy and safety comparison of liraglutide, glimepiride, and placebo, all in combination with metformin, in type 2 diabetes: the LEAD (liraglutide effect and action in diabetes)-2 study. Diabetes Care 32: 84-90. 10.2337/dc08-1355

[13] Zinman B, Gerich J, Buse JB, et al. (2009) Efficacy and safety of the human glucagon-like peptide-1 analog liraglutide in combination with metformin and thiazolidinedione in patients with type 2 diabetes (LEAD-4 Met+TZD). Diabetes Care 32: 1224-1230. 10.2337/dc08-2124

[14] Russell-Jones D, Vaag A, Schmitz O, et al. (2009) Liraglutide vs insulin glargine and placebo in combination with metformin and sulfonylurea therapy in type 2 diabetes mellitus (LEAD-5 met+SU): a randomised controlled trial. Diabetologia 52: 2046-2055

[15] Blonde L, Belousova L, Fainberg U, et al. (2020) Liraglutide as add-on to sodium-glucose co-transporter-2 inhibitors in patients with inadequately controlled type 2 diabetes: LIRA-ADD2SGLT2i, a 26-week, randomized, double-blind, placebo-controlled trial. Diabetes Obes Metab 22: 929-937. 10.1111/dom.13978

[16] Gadde KM, Vetter ML, Iqbal N, Hardy E, Ohman P, investigators D-N-s (2017) Efficacy and safety of autoinjected exenatide once-weekly suspension versus sitagliptin or placebo with metformin in patients with type 2 diabetes: The DURATION-NEO-2 randomized clinical study. Diabetes Obes Metab 19: 979-988. 10.1111/dom.12908

[17] Guja C, Frias JP, Somogyi A, et al. (2018) Effect of exenatide QW or placebo, both added to titrated insulin glargine, in uncontrolled type 2 diabetes: The DURATION-7 randomized study. Diabetes Obes Metab 20: 1602-1614. 10.1111/dom.13266

[18] Wysham C, Blevins T, Arakaki R, et al. (2014) Efficacy and safety of dulaglutide added onto pioglitazone and metformin versus exenatide in type 2 diabetes in a randomized controlled trial (AWARD-1). Diabetes Care 37: 2159-2167. 10.2337/dc13-2760

[19] Skrivanek Z, Gaydos BL, Chien JY, et al. (2014) Dose-finding results in an adaptive, seamless, randomized trial of once-weekly dulaglutide combined with metformin in type 2 diabetes patients (AWARD-5). Diabetes Obes Metab 16: 748-756. 10.1111/dom.12305

[20] Dungan KM, Weitgasser R, Perez Manghi F, et al. (2016) A 24-week study to evaluate the efficacy and safety of once-weekly dulaglutide added on to glimepiride in type 2 diabetes (AWARD-8). Diabetes Obes Metab 18: 475-482. 10.1111/dom.12634

[21] Pozzilli P, Norwood P, Jodar E, et al. (2017) Placebo-controlled, randomized trial of the addition of once-weekly glucagon-like peptide-1 receptor agonist dulaglutide to titrated daily insulin glargine in patients with type 2 diabetes (AWARD-9). Diabetes Obes Metab 19: 1024-1031. 10.1111/dom.12937

[22] Ludvik B, Frias JP, Tinahones FJ, et al. (2018) Dulaglutide as add-on therapy to SGLT2 inhibitors in patients with inadequately controlled type 2 diabetes (AWARD-10): a 24-week, randomised, double-blind, placebo-controlled trial. Lancet Diabetes Endocrinol 6: 370-381. 10.1016/S2213-8587(18)30023-8

[23] Reusch J, Stewart MW, Perkins CM, et al. (2014) Efficacy and safety of once-weekly glucagon-like peptide 1 receptor agonist albiglutide (HARMONY 1 trial): 52-week primary endpoint results from a randomized, double-blind, placebo-controlled trial in patients with type 2 diabetes mellitus not controlled on pioglitazone, with or without metformin. Diabetes Obes Metab 16: 1257-1264. 10.1111/dom.12382

[24] Nauck MA, Stewart MW, Perkins C, et al. (2016) Efficacy and safety of once-weekly GLP-1 receptor agonist albiglutide (HARMONY 2): 52 week primary endpoint results from a randomised, placebo-controlled trial in patients with type 2 diabetes mellitus inadequately controlled with diet and exercise. Diabetologia 59: 266-274. 10.1007/s00125-015-3795-1

[25] Åhren B, Johnson SL, Stewart M, et al. (2014) HARMONY 3: 104-week randomized, double-blind, placebo- and active-controlled trial assessing the efficacy and safety of albiglutide compared with placebo, sitagliptin, and glimepiride in patients with type 2 diabetes taking metformin. Diabetes Care 37: 2141-2148. 10.2337/dc14-0024

[26] Home PD, Shamanna P, Stewart M, et al. (2015) Efficacy and tolerability of albiglutide versus placebo or pioglitazone over 1 year in people with type 2 diabetes currently taking metformin and glimepiride: HARMONY 5. Diabetes Obes Metab 17: 179-187. 10.1111/dom.12414

[27] Sorli C, Harashima SI, Tsoukas GM, et al. (2017) Efficacy and safety of once-weekly semaglutide monotherapy versus placebo in patients with type 2 diabetes (SUSTAIN 1): a double-blind, randomised, placebo-controlled, parallel-group, multinational, multicentre phase 3a trial. Lancet Diabetes Endocrinol 5: 251-260. 10.1016/S2213-8587(17)30013-X

[28] Rodbard HW, Lingvay I, Reed J, et al. (2018) Semaglutide added to basal insulin in type 2 diabetes (SUSTAIN 5): A randomized, controlled trial. J Clin Endocrinol Metab 103: 2291-2301. 10.1210/jc.2018-00070

[29] Aroda VR, Rosenstock J, Terauchi Y, et al. (2019) PIONEER 1: Randomized clinical trial of the efficacy and safety of oral semaglutide monotherapy in comparison with placebo in patients with type 2 diabetes. Diabetes Care 42: 1724-1732. 10.2337/dc19-0749

[30] Pratley R, Amod A, Hoff ST, et al. (2019) Oral semaglutide versus subcutaneous liraglutide and placebo in type 2 diabetes (PIONEER 4): a randomised, double-blind, phase 3a trial. Lancet 394: 39-50. 10.1016/S0140-6736(19)31271-1

[31] Mosenzon O, Blicher TM, Rosenlund S, et al. (2019) Efficacy and safety of oral semaglutide in patients with type 2 diabetes and moderate renal impairment (PIONEER 5): a placebo-controlled, randomised, phase 3a trial. Lancet Diabetes Endocrinol 7(7): 515-527. 10.1016/S2213-8587(19)30192-5

[32] Zinman B, Aroda VR, Buse JB, et al. (2019) Efficacy, safety, and tolerability of oral semaglutide versus placebo added to insulin with or without metformin in patients with type 2 diabetes: The PIONEER 8 trial. Diabetes Care 42: 2262-2271. 10.2337/dc19-0898

[33] Yamada Y, Katagiri H, Hamamoto Y, et al. (2020) Dose-response, efficacy, and safety of oral semaglutide monotherapy in Japanese patients with type 2 diabetes (PIONEER 9): a 52-week, phase 2/3a, randomised, controlled trial. Lancet Diabetes Endocrinol 8: 377-391. 10.1016/S2213-8587(20)30075-9

[34] Rosenstock J, Wysham C, Frias JP, et al. (2021) Efficacy and safety of a novel dual GIP and GLP-1 receptor agonist tirzepatide in patients with type 2 diabetes (SURPASS-1): a double-blind, randomised, phase 3 trial. Lancet 398: 143-155. 10.1016/S0140-6736(21)01324-6

[35] Dahl D, Onishi Y, Norwood P, et al. (2022) Effect of subcutaneous tirzepatide vs placebo added to titrated insulin glargine on glycemic control in patients with type 2 diabetes: The SURPASS-5 randomized clinical trial. JAMA 327: 534-545. 10.1001/jama.2022.0078

[36] Linnebjerg H, Kothare PA, Park S, et al. (2007) Effect of renal impairment on the pharmacokinetics of exenatide. Br J Clin Pharmacol 64(3): 317-327

[37] Kolterman OG, Kim DD, Shen L, et al. (2005) Pharmacokinetics, pharmacodynamics, and safety of exenatide in patients with type 2 diabetes mellitus. Am J Health Syst Pharm 62(2): 173-181. 10.1093/ajhp/62.2.173

[38] Fineman MS, Bicsak TA, Shen LZ, et al. (2003) Effect on glycemic control of exenatide (synthetic exendin-4) additive to existing metformin and/or sulfonylurea treatment in patients with type 2 diabetes. Diabetes Care 26: 2370-2377

[39] Ratner RE, Rosenstock J, Boka G, Investigators DRIS (2010) Dose-dependent effects of the once-daily GLP-1 receptor agonist lixisenatide in patients with Type 2 diabetes inadequately controlled with metformin: a randomized, double-blind, placebo-controlled trial. Diabet Med 27: 1024-1032. 10.1111/j.1464-5491.2010.03020.x

[40] Agersø H, Jensen LB, Elbrønd B, Rolan P, Zdravkovic M (2002) The pharmacokinetics, pharmacodynamics, safety and tolerability of NN2211, a new long-acting GLP-1 derivative, in healthy men. Diabetologia 45: 195-202. 10.1007/s00125-001-0719-z

[41] Flint A, Nazzal K, Jagielski P, Hindsberger C, Zdravkovic M (2010) Influence of hepatic impairment on pharmacokinetics of the human GLP-1 analogue, liraglutide. Br J Clin Pharmacol 70: 807-814. 10.1111/j.1365-2125.2010.03762.x

[42] Jacobsen LV, Hindsberger C, Robson R, Zdravkovic M (2009) Effect of renal impairment on the pharmacokinetics of the GLP-1 analogue liraglutide. Br J Clin Pharmacol 68(6): 898-905. 10.1111/j.1365-2125.2009.03536.x

[43] Madsbad S, Schmitz O, Ranstam J, Jakobsen G, Matthews DR, Group NNIS (2004) Improved glycemic control with no weight increase in patients with type 2 diabetes after once-daily treatment with the long-acting glucagon-like peptide 1 analog liraglutide (NN2211): a 12-week, double-blind, randomized, controlled trial. Diabetes Care 27: 1335-1342. 10.2337/diacare.27.6.1335

[44] Nauck MA, Hompesch M, Filipczak R, et al. (2006) Five weeks of treatment with the GLP-1 analogue liraglutide improves glycaemic control and lowers body weight in subjects with type 2 diabetes. Exp Clin Endocrinol Diabetes 114: 417-423. 10.1055/s-2006-924230

[45] Feinglos MN, Saad MF, Pi-Sunyer FX, An B, Santiago O, Liraglutide Dose-Response Study G (2005) Effects of liraglutide (NN2211), a long-acting GLP-1 analogue, on glycaemic control and bodyweight in subjects with Type 2 diabetes. Diabet Med 22: 1016-1023. 10.1111/j.1464-5491.2005.01567.x

[46] Fineman M, Flanagan S, Taylor K, et al. (2011) Pharmacokinetics and pharmacodynamics of exenatide extended-release after single and multiple dosing. Clin Pharmacokinet 50: 65-74. 10.2165/11585880-000000000-00000

[47] Kim D, MacConell L, Zhuang D, et al. (2007) Effects of once-weekly dosing of a long-acting release formulation of exenatide on glucose control and body weight in subjects with type 2 diabetes. Diabetes Care 30: 1487-1493

[48] Cui YM, Guo XH, Zhang DM, et al. (2013) Pharmacokinetics, safety, and tolerability of single- and multiple-dose exenatide once weekly in Chinese patients with type 2 diabetes mellitus. J Diabetes 5(2): 127-135. 10.1111/1753-0407.12020

[49] Iwamoto K, Nasu R, Yamamura A, et al. (2009) Safety, tolerability, pharmacokinetics, and pharmacodynamics of exenatide once weekly in Japanese patients with type 2 diabetes. Endocr J 56: 951-962. 10.1507/endocrj.k09e-147

[50] Barrington P, Chien JY, Showalter HD, et al. (2011) A 5-week study of the pharmacokinetics and pharmacodynamics of LY2189265, a novel, long-acting glucagon-like peptide-1 analogue, in patients with type 2 diabetes. Diabetes Obes Metab 13: 426-433. 10.1111/j.1463-1326.2011.01364.x

[51] Umpierrez GE, Blevins T, Rosenstock J, et al. (2011) The effects of LY2189265, a long-acting glucagon-like peptide-1 analogue, in a randomized, placebo-controlled, double-blind study of overweight/obese patients with type 2 diabetes: the EGO study. Diabetes Obes Metab 13: 418-425. 10.1111/j.1463-1326.2011.01366.x

[52] Xu J, Zhang Y, Li Y, et al. (2022) Pharmacokinetics, pharmacodynamics, and safety of dulaglutide after single or multiple doses in Chinese healthy subjects and patients with T2DM: A randomized, placebo-controlled, phase I study. Adv Ther 39: 488-503. 10.1007/s12325-021-01921-5

[53] Grunberger G, Chang A, Garcia Soria G, Botros FT, Bsharat R, Milicevic Z (2012) Monotherapy with the once-weekly GLP-1 analogue dulaglutide for 12 weeks in patients with Type 2 diabetes: dose-dependent effects on glycaemic control in a randomized, double-blind, placebo-controlled study. Diabet Med 29: 1260-1267. 10.1111/j.1464-5491.2012.03745.x

[54] Terauchi Y, Satoi Y, Takeuchi M, Imaoka T (2014) Monotherapy with the once weekly GLP-1 receptor agonist dulaglutide for 12 weeks in Japanese patients with type 2 diabetes: dose-dependent effects on glycaemic control in a randomised, double-blind, placebo-controlled study. Endocr J 61: 949-599. 10.1507/endocrj.ej14-0147

[55] Shaddinger BC, Vlasakakis G, Soffer J, Thorpe KM, Hatch D, Nino AJ (2019) A randomized, double-blind, single-dose, crossover study to demonstrate the bioequivalence of 2 formulations of albiglutide in healthy adult participants. Clin Pharmacol Drug Dev 8: 361-370. 10.1002/cpdd.606

[56] Matthews JE, Stewart MW, De Boever EH, et al. (2008) Pharmacodynamics, pharmacokinetics, safety, and tolerability of albiglutide, a long-acting glucagon-like peptide-1 mimetic, in patients with type 2 diabetes. J Clin Endocrinol Metab 93: 4810-4817. 10.1210/jc.2008-1518

[57] Young MA, Wald JA, Matthews JE, Yang F, Reinhardt RR (2014) Effect of renal impairment on the pharmacokinetics, efficacy, and safety of albiglutide. Postgrad Med 126(3): 35-46. 10.3810/pgm.2014.05.2754

[58] Rosenstock J, Reusch J, Bush M, Yang F, Stewart M, Albiglutide Study G (2009) Potential of albiglutide, a long-acting GLP-1 receptor agonist, in type 2 diabetes: a randomized controlled trial exploring weekly, biweekly, and monthly dosing. Diabetes Care 32: 1880-6188. 10.2337/dc09-0366

[59] Seino Y, Nakajima H, Miyahara H, et al. (2009) Safety, tolerability, pharmacokinetics and pharmacodynamics of albiglutide, a long-acting GLP-1-receptor agonist, in Japanese subjects with type 2 diabetes mellitus. Curr Med Res Opin 25(12): 3049-3057

[60] Marbury TC, Flint A, Jacobsen JB, Derving Karsbol J, Lasseter K (2017) Pharmacokinetics and tolerability of a single dose of semaglutide, a human glucagon-like peptide-1 analog, in subjects with and without renal impairment. Clin Pharmacokinet 56: 1381-1390. 10.1007/s40262-017-0528-2

[61] Jensen L, Kupcova V, Arold G, Pettersson J, Hjerpsted JB (2018) Pharmacokinetics and tolerability of semaglutide in people with hepatic impairment. Diabetes Obes Metab 20: 998-1005. 10.1111/dom.13186

[62] Kapitza C, Nosek L, Jensen L, Hartvig H, Jensen CB, Flint A (2015) Semaglutide, a once-weekly human GLP-1 analog, does not reduce the bioavailability of the combined oral contraceptive, ethinylestradiol/levonorgestrel. J Clin Pharmacol 55: 497-504. 10.1002/jcph.443

[63] Nauck MA, Petrie JR, Sesti G, et al. (2016) A phase 2, randomized, dose-finding study of the novel Once-weekly human GLP-1 analog, semaglutide, compared with placebo and open-label liraglutide in patients with type 2 diabetes. Diabetes Care 39: 231-241. 10.2337/dc15-0165

[64] Baekdal TA, Thomsen M, Kupcova V, Hansen CW, Anderson TW (2018) Pharmacokinetics, safety, and tolerability of oral semaglutide in subjects with hepatic impairment. J Clin Pharmacol 58: 1314-1323. 10.1002/jcph.1131

[65] Granhall C, Sondergaard FL, Thomsen M, Anderson TW (2018) Pharmacokinetics, safety and tolerability of oral semaglutide in subjects with renal impairment. Clin Pharmacokinet 57: 1571-1580. 10.1007/s40262-018-0649-2

[66] van Hout M, Forte P, Jensen TB, Boschini C, Baekdal TA (2023) Effect of various dosing schedules on the pharmacokinetics of oral semaglutide: A randomised trial in healthy subjects. Clin Pharmacokinet 62: 635-644. 10.1007/s40262-023-01223-9

[67] Davies M, Pieber TR, Hartoft-Nielsen ML, Hansen OKH, Jabbour S, Rosenstock J (2017) Effect of oral semaglutide compared with placebo and subcutaneous semaglutide on glycemic control in patients with type 2 diabetes: A randomized clinical trial. J Am Med Ass 318: 1460-1470. 10.1001/jama.2017.14752

[68] Coskun T, Sloop KW, Loghin C, et al. (2018) fuLY3298176, a novel dual GIP and GLP-1 receptor agonist for the treatment of type 2 diabetes mellitus: From discovery to clinical proof of concept. Mol Metab 18: 3-14. 10.1016/j.molmet.2018.09.009

[69] Furihata K, Mimura H, Urva S, Oura T, Ohwaki K, Imaoka T (2022) A phase 1 multiple-ascending dose study of tirzepatide in Japanese participants with type 2 diabetes. Diabetes Obes Metab 24: 239-246. 10.1111/dom.14572

[70] Frias JP, Nauck MA, Van J, et al. (2020) Efficacy and tolerability of tirzepatide, a dual glucose-dependent insulinotropic peptide and glucagon-like peptide-1 receptor agonist in patients with type 2 diabetes: A 12-week, randomized, double-blind, placebo-controlled study to evaluate different dose-escalation regimens. Diabetes Obes Metab 22: 938-946. 10.1111/dom.13979

[71] Kolterman OG, Kim DD, Shen L, et al. (2005) Pharmacokinetics, pharmacodynamics, and safety of exenatide in patients with type 2 diabetes mellitus. Am J Health Syst Pharm 62: 173-181. 10.1093/ajhp/62.2.173

[72] Barrientos-Perez M, Hsia DS, Sloan L, et al. (2022) A study on pharmacokinetics, pharmacodynamics and safety of lixisenatide in children and adolescents with type 2 diabetes. Pediatr Diabetes 23: 641-648. 10.1111/pedi.13343

[73] Petri KC, Jacobsen LV, Klein DJ (2015) Comparable liraglutide pharmacokinetics in pediatric and adult populations with type 2 diabetes: a population pharmacokinetic analysis. Clin Pharmacokinet 54: 663-670. 10.1007/s40262-014-0229-z

[74] Li H, Xu J, Fan X (2015) Target-mediated pharmacokinetic/pharmacodynamic model based meta-analysis and dosing regimen optimization of a long-acting release formulation of exenatide in patients with type 2 diabetes mellitus. J Pharmacol Sci 127: 170-180. 10.1016/j.jphs.2014.12.004

[75] Cui YM, Guo XH, Zhang DM, et al. (2013) Pharmacokinetics, safety, and tolerability of single- and multiple-dose exenatide once weekly in Chinese patients with type 2 diabetes mellitus. J Diabetes 5: 127-135. 10.1111/1753-0407.12020

[76] Geiser JS, Heathman MA, Cui X, et al. (2016) Clinical pharmacokinetics of dulaglutide in patients with type 2 diabetes: Analyses of data from clinical trials. Clin Pharmacokinet 55: 625-634. 10.1007/s40262-015-0338-3

[77] Blair HA, Keating GM (2015) Albiglutide: a review of its use in patients with type 2 diabetes mellitus. Drugs 75: 651-663. 10.1007/s40265-015-0370-5

[78] Overgaard RV, Delff PH, Petri KCC, Anderson TW, Flint A, Ingwersen SH (2019) Population pharmacokinetics of semaglutide for type 2 diabetes. Diabetes Ther 10: 649-662. 10.1007/s13300-019-0581-y

[79] Carlsson Petri KC, Ingwersen SH, Flint A, Zacho J, Overgaard RV (2018) Semaglutide s.c. once-weekly in type 2 diabetes: A population pharmacokinetic analysis. Diabetes Ther 9: 1533-1547. 10.1007/s13300-018-0458-5

[80] Ikushima I, Jensen L, Flint A, Nishida T, Zacho J, Irie S (2018) A randomized trial investigating the pharmacokinetics, pharmacodynamics, and safety of subcutaneous semaglutide once-weekly in healthy male Japanese and Caucasian subjects. Adv Ther 35: 531-544. 10.1007/s12325-018-0677-1

[81] Granhall C, Donsmark M, Blicher TM, et al. (2019) Safety and pharmacokinetics of single and multiple ascending doses of the novel oral human GLP-1 analogue, oral semaglutide, in healthy subjects and subjects with type 2 diabetes. Clin Pharmacokinet 58: 781-791. 10.1007/s40262-018-0728-4

[82] Schneck K, Urva S (2024) Population pharmacokinetics of the GIP/GLP receptor agonist tirzepatide. CPT Pharmacometrics Syst Pharmacol 13: 494-503. 10.1002/psp4.13099
